# Contribution of the *nos-pdt* Operon to Virulence Phenotypes in Methicillin-Sensitive *Staphylococcus aureus*

**DOI:** 10.1371/journal.pone.0108868

**Published:** 2014-10-02

**Authors:** April M. Sapp, Austin B. Mogen, Erin A. Almand, Frances E. Rivera, Lindsey N. Shaw, Anthony R. Richardson, Kelly C. Rice

**Affiliations:** 1 Department of Microbiology and Cell Science, University of Florida, Gainesville, Florida, United States of America; 2 Department of Microbiology, North Carolina State University, Raleigh, North Carolina, United States of America; 3 Department of Cell Biology, Microbiology and Molecular Biology, University of South Florida, Tampa, Florida, United States of America; 4 Department of Microbiology and Immunology, University of North Carolina at Chapel Hill, Chapel Hill, North Carolina, United States of America; University of Illinois at Chicago College of Medicine, United States of America

## Abstract

Nitric oxide (NO) is emerging as an important regulator of bacterial stress resistance, biofilm development, and virulence. One potential source of endogenous NO production in the pathogen *Staphylococcus aureus* is its NO-synthase (saNOS) enzyme, encoded by the *nos* gene. Although a role for saNOS in oxidative stress resistance, antibiotic resistance, and virulence has been recently-described, insights into the regulation of *nos* expression and saNOS enzyme activity remain elusive. To this end, transcriptional analysis of the *nos* gene in *S. aureus* strain UAMS-1 was performed, which revealed that *nos* expression increases during low-oxygen growth and is growth-phase dependent. Furthermore, *nos* is co-transcribed with a downstream gene, designated *pdt*, which encodes a prephenate dehydratase (PDT) enzyme involved in phenylalanine biosynthesis. Deletion of *pdt* significantly impaired the ability of UAMS-1 to grow in chemically-defined media lacking phenylalanine, confirming the function of this enzyme. Bioinformatics analysis revealed that the operon organization of *nos-pdt* appears to be unique to the staphylococci. As described for other *S. aureus nos* mutants, inactivation of *nos* in UAMS-1 conferred sensitivity to oxidative stress, while deletion of *pdt* did not affect this phenotype. The *nos* mutant also displayed reduced virulence in a murine sepsis infection model, and increased carotenoid pigmentation when cultured on agar plates, both previously-undescribed *nos* mutant phenotypes. Utilizing the fluorescent stain 4-Amino-5-Methylamino-2',7'-Difluorofluorescein (DAF-FM) diacetate, decreased levels of intracellular NO/reactive nitrogen species (RNS) were detected in the *nos* mutant on agar plates. These results reinforce the important role of saNOS in *S. aureus* physiology and virulence, and have identified an *in vitro* growth condition under which saNOS activity appears to be upregulated. However, the significance of the operon organization of *nos-pdt* and potential relationship between these two enzymes remains to be elucidated.

## Introduction

Nitric Oxide (NO) is a highly reactive free radical gas that easily and rapidly diffuses across biological membranes [1], [2]. One cellular source of NO production is via the NO synthase enzymes (NOS), whose three isoforms are well-characterized in mammals and are involved in a wide variety of cellular processes, including regulation of blood pressure, nervous system signaling, and protection against pathogens [3]–[9]. All three mammalian NOS enzymes consist of both an oxygenase and reductase domain, which together catalyze a five-electron heme-based oxidation of the guanidine nitrogen of L-arginine to L-citrulline [7]. The power and versatility of NO as a signaling molecule is exemplified by its ability to diffuse several cell diameters away from its site of synthesis in eukaryotes [1], [10]. NO can react directly or indirectly via formation of reactive nitrogen species (RNS) with a variety of cellular targets, including DNA, lipids, and proteins containing transition metal centers and thiol groups (reviewed in [11]).

A number of studies in recent years have revealed that some bacterial species also contain a NOS homologue in their genomes, equivalent in most cases to the oxygenase domain of eukaryotic NOS (reviewed in [3]). The role of bacterial NOS can be quite varied, participating in diverse physiological functions such as biofilm development, biosynthesis of plant phytotoxins, stress resistance and virulence [12]–[18]. Although this versatility suggests a specificity of function to individual bacterial species, an increased understanding of the regulation and exact cellular targets of bacterial NOS may help divulge an evolutionary link between prokaryotic and eukaryotic organisms. Previous analysis of bacterial genomes has shown that bacterial NOS enzymes are primarily found in Gram-positive species such as *Streptomyces*, *Bacillus*, and the staphylococci [3]. A number of biochemical studies have focused on determining the crystal structure and characterizing the enzymatic activity of these bacterial NOS enzymes (reviewed in [4]). By comparison, much less is understood about the regulation of bacterial NOS enzyme expression, activity, and participation in cell signaling.

*Staphylococcus aureus* is a versatile pathogen that is highly transmissible in healthcare settings as well as in the community at-large. Of increasing concern is the fact that many methicillin-resistant *S. aureus* (MRSA) strains are genetically-resistant to nearly all antibiotics currently used in the clinical setting. In fact, a recent report by the CDC estimates that 80,000 MRSA infections and 11,000 MRSA-associated deaths occur in the US per year [19]. These alarming statistics underscore the importance of deciphering new drug targets to expand our arsenal against this devastating pathogen. Bacterial NOS enzymes are an attractive target for drug development, since they have been shown to confer tolerance to oxidative stress [14], [15], [20] and antibiotics [18], [21], promote bacterial survival inside neutrophils [20] and macrophages [14], [21], and contribute to virulence *in vivo* [14], [21]. A search for small molecule inhibitors of *Bacillus subtilis* NOS has also recently been undertaken and described [22]. With respect to *S. aureus*, exogenous addition of NO to cultures protects this bacterium from challenge with H_2_O_2_ and antimicrobials [15], [18]. More recent studies of *S. aureus nos* mutants have revealed that this gene contributes to a number of virulence properties, including protection against oxidative stress [20], [21], antibiotics [21], antimicrobial peptides [21], and leucocyte-killing [20], [21]. A role for saNOS in *S. aureus* virulence was also recently confirmed in a murine abscess model of infection [21]. Although multiple models infer the importance of bacterial NOS during infection, the exact molecular mechanisms by which NOS elicits its control on bacterial stress resistance and virulence have not been elucidated.

Given the established relevance of saNOS in virulence and as a potential drug development target [22], it is surprising that relatively little attention has been given to the genetics, regulation, and the exact cellular targets of this enzyme. Inspection of the *S. aureus* genome has revealed that the *nos* open reading frame (ORF; SAR2007 of the MRSA252 genome) is separated by only 19 nucleotides from the downstream SAR2008 ORF. No identifiable transcription termination signals appear to be present between *nos* and SAR2008, suggesting that they may comprise an operon. SAR2008 (henceforth referred to as the *pdt* gene) encodes a prephenate dehydratase (saPDT) enzyme, which catalyzes the formation of phenylpyruvate from prephenate during phenylalanine biosynthesis [23]. Therefore, this current study was undertaken to probe the potential genetic and functional relationships between saNOS and saPDT in a clinical methicillin-sensitive *S. aureus* strain. These experiments demonstrate that the *nos* and *pdt* genes are co-transcribed, and that the expression of this operon is upregulated during low-oxygen growth. Furthermore, a *nos*::*erm* mutant displayed reduced virulence in a murine sepsis model and increased carotenoid pigmentation when cultured on agar plates, both previously-undescribed *nos* mutant phenotypes. An assay to detect intracellular NO/RNS in *S. aureus* was also developed using the fluorescent stain DAF-FM diacetate, and demonstrated decreased levels of intracellular NO/RNS in the *nos* mutant when cultured on agar plates.

## Materials and Methods

### Bacterial strains and growth conditions

*Escherichia coli* and *Staphylococcus aureus* strains and plasmids used in this study are listed in Table 1. Planktonic *S. aureus* cultures were grown either aerobically (1∶10 volume to flask ratio, 250 RPM) or under low-oxygen conditions (7∶10 volume to flask ratio, 0 RPM), as indicated for each experiment. *S. aureus* was freshly streaked from a frozen stock onto tryptic soy agar (TSA) for 24 hours, followed by overnight aerobic growth in tryptic soy broth (TSB) for each experiment, unless otherwise indicated. As indicated, antibiotics were included in agar plates and broth cultures at the following concentrations: 5 µg/ml or 10 µg/ml chloramphenicol (Cm), 2 µg/ml or 10 µg/ml erythromycin (Erm). *E. coli* was routinely grown under aerobic conditions (1∶10 volume to flask ratio, 250 RPM) in Luria-Bertani (LB) broth with 50 µg/ml ampicillin (Amp) or 50 µg/ml kanamycin (Km), as indicated in Table 1. Glycerol stock cultures of all strains were maintained at −80°C and were prepared by mixing equal volume of overnight culture with sterile 50% (vol/vol) glycerol in cryogenic tubes.

**Table 1:** Bacterial strains and plasmid constructs used in this study.

Strain or plasmid name	Description	Reference or source
*Escherichia coli*		
DH5α	Host strain for construction of recombinant plasmids	[68]
*Staphylococcus aureus*		
RN4220	Easily transformable restriction deficient strain	[69]
UAMS-1	Osteomyelitis clinical isolate	[70]
KR1010	UAMS-1 *nos*::*erm* insertion mutant	This work
KR1013	UAMS-1 Δ*pdt* deletion mutant	This work
Plasmids		
pTR27	*nos*::*erm* allele-replacement plasmid; Erm^R^/Cm^R^	This work
pKR13	Δ*pdt* allele-replacement plasmid; Cm^R^	This work
pMKnos	*nos* complementation plasmid; Cm^R^/Amp^R^	This work
pMKpdt	*pdt* complementation plasmid; Cm^R^/Amp^R^	This work
pCRBlunt	*E. coli* cloning plasmid; Km^R^	Invitrogen
pCR2.1	*E. coli* cloning plasmid; Km^R^/Amp^R^	Invitrogen
pBSK	*E. coli* cloning plasmid; Amp^R^	Stratagene
pBT2	Temperature-sensitive shuttle vector; Cm^R^/Amp^R^	[25]
pMK4	Shuttle vector; Cm^R^/Amp^R^	[29]

### Creation of *nos*::*erm* and Δ*pdt* mutant strains

To create a *nos* **::** *erm* insertion mutant, plasmid pTR27 (Table 1) was created as follows: The *nos* gene was amplified from *S. aureus* genomic DNA by PCR using the nos1-F and nos1-R primers specified in Table 2, followed by Topo-cloning into pCR2.1 (Invitrogen). An internal **BglII** site was introduced into this cloned sequence 232 bp downstream from the saNOS start codon in the sequence 5'-gttaaatgtcattgatgcaagAGATGTtactgacgaagcatcgttcttatc-3', where the capital letters are located, changing the sequence from AGATGT to AGATCT, a **BglII** restriction enzyme cut site. An *Eco*RI fragment harboring this modified allele was moved into the **Eco**RI site of pBluescript SK (pBSK), a vector backbone that does not contain a **Bgl**II site. A 1.1 kb **Bam**HI-digested fragment harboring the ***erm*** resistance cassette from Tn1545 [24] was then cloned into the **Bgl**II site of the *nos* fragment cloned in pBSK. The resulting *nos* **::** *erm* allele was subsequently ligated into the **Eco**RI site of the temperature sensitive shuttle vector pBT2 [25] to generate pTR27, which was first transformed into strain RN4220 by electroporation, followed by phage transduction into UAMS-1, with growth at 30°C for all steps [26]–[28]. To create strain KR1010 (UAMS-1 *nos* **::** *erm* mutant), integration of pTR27 into the *nos* gene on the UAMS-1 chromosome was initiated by growth at 43°C on TSA +10 µg/ml Erm (non-permissive temperature for plasmid replication), to promote integration of the plasmid into the chromosome via homologous recombination at the *nos* gene. To induce a second recombination event, a single isolated colony was used to inoculate TSB (no antibiotic) and grown at 30°C for 5 days. Every 24 hours, an aliquot of the culture was diluted 1000-fold into fresh TSB (no antibiotic). On days 3–5, the culture was serially-diluted and spread on TSA +10 µg/ml Erm, and isolated colonies were then screened for Erm-resistant and Cm-sensitive phenotypes by picking and patching onto TSA +2 µg/ml Erm and TSA +5 µg/ml Cm. PCR using the nos1-F and nos1-R primer pair, as well as Southern blotting, were used to screen candidate mutants to confirm that the chromosomal *nos* gene had been correctly replaced by the *nos* **::** *erm* allele.

**Table 2:** PCR primers used in this study.

Primer	Purpose	Sequence (5′-3′; lower case letters represent addition of restriction site)
nos1-F nos1-R	*nos*::*erm* mutant creation	GTTAGTTACCAAAGCATAATTGCG ATCTGCCACAATGTTGATTGTTCC
nos2-F nos2-R	*nos* complement	cccggatccGTTTCTATTAATTGCGCTTGA cccgaattcTGCTATACCTCTACTAACTTAATGAT
nos3-F nos3-R	*nos* promoter	cccggatccTGTTTCTATTAATTGCGCTTGAA ccccccgggGCTTTATATTATAGTCTAC
nos4-F nos4-R	real-time PCR	TATGGTGCTAAAATGGCTTG ACGATGCTTCGTCAGTAACA
nos5-F pdt5-R	co-transcription PCR	ccccatatgTTATTTAAAGAGGCTCAAGCTTTC cccctcgagATTAAAAGCACCAATCA
pdt1-F pdt1-R	*Δpdt* mutant creation	TTTAAAGAGGCTCAAGCTTTCA CAACACCAATCGATGTGTCA
pdt2-F pdt2-R	*Δpdt* mutant creation	CAATTAGGTATGTATCGATTTTTCG ccctctagaAAACAAAAGTTAGACGAGCTTGG
pdt3-F pdt3-R	*pdt* complement	ccccccgggCATCATTAAGTTAGTAGAGGTATAGCA cccgaattcACGAGTGGTAACGAAACGTA
pdt4-F pdt4-R	real-time PCR	TGGGTTGTTAGCAAGTGTGC GCCTGAACGAAAAATCGATAC
sigA-F sigA-R	real-time PCR	CAAGCAATCACTCGTGCAAT GGTGCTGGATCTCGACCTAA
asp23-F asp23-R	real-time PCR	ATTGCTGGTATCGCTGCAC TTGTTGCCACTTGAGAATGC
purH-F purH-R	real-time PCR	CGAAATAAACCGCAGCATTT TCGTCACATCAGGGTTAGCA
crtN-F crtN-R	real-time PCR	CAGTGATTGGTGCAGGTGTC CATACGCCCGCCTACATTAT

To create a Δ*pdt* deletion mutant, plasmid pKR13 (Table 1) was created as follows: PCR was performed with primer pairs pdt1-F/pdt1-R (amplifying the 1.2-kb region upstream of nucleotide 151 of the *pdt* open reading frame), and pdt2-F/pdt2-R (amplifying the 1.0-kb region downstream of nucleotide 672 of the *pdt* ORF) using AccuPrime™ Pfx SuperMix (Invitrogen Life Technologies) and UAMS-1 genomic DNA as template. Each PCR product was cloned into pCRBlunt (Invitrogen Life Technologies) following the manufacturer's protocols for ligation and *E. coli* transformation, followed by Sanger DNA sequencing (ICBR, University of Florida) to verify that no mutations had been introduced. The pdt1 and pdt2 inserts were each excised from pCRBlunt by restriction digestion with HindIII-ClaI and ClaI-XbaI, respectively, followed by gel-purification of each insert (DNA Gel Extraction Kit, Zymo Research). Plasmid pBT2 was digested with HindIII-XbaI, and a “triple” ligation reaction using T4 DNA ligase (New England Biolabs) containing this linearized plasmid, HindIII-ClaI pdt1 and ClaI-XbaI pdt2 was incubated for several hours at room temperature, followed by heat-shock transformation into *E. coli* DH5α. The resulting pKR13 was moved into *S. aureus* RN4220 and UAMS-1 by electroporation and phage transduction, respectively, as described above. Creation of strain KR1013 (UAMS-1 Δ*pdt* mutant) was performed as described above, except that isolated colonies recovered after induction of the second recombination event were screened for chloramphenicol sensitivity by picking and patching onto TSA (no antibiotic) and TSA +5 µg/ml Cm. PCR with primers nos2-F and pdt2-R was used to screen candidate mutants to confirm that 521-bp of the *pdt* gene had been deleted.

### Creation of complementation plasmids

To create pMKnos, primer pair nos2-F/nos2-R and Thermalace enzyme (Invitrogen Life Sciences) was used to PCR-amplify a 1.6 kb product (spanning 564 nucleotides upstream of the *nos* ATG start codon through the entire *nos* ORF), using UAMS-1 genomic DNA as template. This PCR product was cloned into pCRBlunt (Invitrogen Life Technologies) according to the manufacturer's protocols for ligation and *E. coli* transformation, followed by Sanger DNA sequencing to verify that no mutations had been introduced. This DNA fragment was excised from pCRBlunt by restriction digestion with BamHI and EcoRI, gel-purified, and ligated to BamHI-EcoRI digested pMK4 [29]. This ligation was transformed into *E. coli*, followed by movement into *S. aureus* RN4220 and KR1010 by electroporation and phage transduction, respectively, as described above. To create pMKpdt, primer pair nos3-F/nos3-R was used to PCR-amplify a product spanning 564 nucleotides upstream of the *nos* ATG start codon, and primer pair pdt3-F/pdt3-R was used to amplify a 0.97 kb product spanning the *pdt* ORF. Each PCR product was cloned into pCRBlunt (Invitrogen Life Technologies) following the manufacturer's protocols for ligation and *E. coli* transformation, followed by Sanger DNA sequencing. The *nos* promoter fragment was excised from pCRBlunt by restriction digestion with BamHI and SmaI, gel-purified, and ligated to BamHI-SmaI digested pMK4 and transformed into *E. coli*. The resulting plasmid was then digested with SmaI and EcoRI, and ligated to the 0.97-kb *pdt* insert that was excised from pCRBlunt by digestion with these same two enzymes. This ligation was then transformed into *E. coli*, followed by movement into *S. aureus* RN4220, KR1010, and KR1013 by electroporation and phage transduction, respectively, as described above. *S. aureus* UAMS-1, KR1010, and KR1013 were also transduced with pMK4 to create vector-only control strains.

### Growth experiments and RNA isolation

To measure growth-phase dependent expression of *nos* and *pdt*, overnight cultures of UAMS-1 were diluted to an OD_600_ = 0.05 in TSB, and were grown in both aerobic and low-oxygen conditions as described above (n = 3 biological replicates). Culture samples were harvested from each flask at 2, 6, and 12 hours growth. To compare *nos* and *pdt* expression between UAMS-1 (pMK4), *nos*::*erm* mutant (pMK4) and *nos*::*erm* complement (pMKnos) strains, cultures of each (n = 3 biological replicates) were grown for 6 hours under low-oxygen conditions. To compare expression of *nos*, *pdt*, and various carotenoid pigment-related genes (Table 2) between UAMS-1 (pMK4), *nos*::*erm* mutant (pMK4), *nos* complement (*nos*::*erm* mutant containing pMKnos) and *pdt* complement (*nos*::*erm* mutant containing pMKpdt) strains, cultures of each (n = 3 biological replicates) were grown for 28 hours on TSA +5 µg/ml Cm. For all experiments, RNA was isolated with the FASTPREP system (Lysing Matrix B, Q-Biogene, Inc.) and Qiagen RNeasy mini kit, using previously-published methods [30].

### Quantitative real-time PCR (qRT-PCR) analysis of gene expression

To remove residual genomic contamination, isolated RNA was treated with Turbo DNase using the TURBO DNA-*free*™ kit (Applied Biosystems). A mock qRT-PCR was then performed with primer pair sigA-F/sigA-R and DNAse-treated RNA as template to check for amplification of contaminating genomic DNA. Each RNA sample (0.750 µg) was then converted to cDNA using the iScript Reverse Transcriptase kit (BioRad), which generates total cDNA using a mixture of oligo(dT) and random hexamer primers. Expression of genes of interest (Table 2) was measured in the cDNA from each sample by qRT-PCR using iQ SYBR green supermix (BioRad) and the Eco Real-Time PCR system (Illumina). The Livak method (2^-ΔΔCt^) [31] was used to calculate the relative fold change between the calibrator (indicated in each figure legend) and test samples. Primers specific to the housekeeping gene sigA (sigA-F/sigA-R) were used to normalize the data. The amplification efficiency of sigA-F/sigA-R was within 5% of all of the other primer pairs used in qRT-PCR. All qRT-PCR reactions were performed on triplicate technical replicates of each RNA isolated from n = 3 biological samples.

### Co-transcription PCR

To determine whether the *nos* and *pdt* genes are co-transcribed, 0.750 µg of UAMS-1 RNA isolated from 6 hour low-oxygen cultures (n = 3 biological replicates) was converted to cDNA using the iScript select cDNA kit (BioRad) and primer pdt5-R, which anneals to the stop codon region of the *pdt* gene. As a control, these reactions were also performed in the absence of reverse-transcriptase (RT) enzyme, to check for genomic DNA contamination. PCR reactions (30 cycles) were then performed on 0.075 µg of each RT+ and RT- cDNA sample, as well as a UAMS-1 genomic DNA template control and no template control. Primer pair nos5-F/pdt5-R was used to amplify a 1.9-kb product that spans the region between the *nos* start codon and *pdt* stop codon. PCR products were visualized by agarose gel electrophoresis and ethidium bromide staining, and imaged on a Molecular Imager Gel Doc XR+ (BioRad).

### Carotenoid pigment assays

The pigment differences in UAMS-1 (pMK4), *nos* **::** *erm* mutant (pMK4), ***nos*** complement (*nos* **::** *erm* mutant containing pMKnos), and ***pdt*** complement (*nos* **::** *erm* mutant containing pMKpdt) strains were quantitatively assessed using a modified method from Morikawa et al. [32]. In brief, each strain was grown at 37°C on TSA +5 µg/ml Cm. After 48 hours growth, cells were scraped from each plate, suspended in 1 ml dH_2_O, and centrifuged for 3 minutes at 13,000 RPM. The supernatants were removed and cells were washed and centrifuged a second time as described above. Cell pellets were then resuspended in 420 µl methanol, vortexed for 10 seconds, and 20 µl was withdrawn to measure the OD_600_ of each suspension. The remaining 400 µl was incubated at 55°C for 5 minutes, and then centrifuged at 13,000 RPM for 2 minutes. Finally, 350 µl supernatant (containing extracted pigments) was withdrawn from each tube, transferred to a cuvette containing 650 µl methanol, and the absorbance of each sample at 465 nm. All samples were standardized by dividing the A_465_ reading by the sample's corresponding relative OD_600_ value [calculated by dividing the OD_600_ of each sample by the average OD_600_ of UAMS-1 (pMK4)], to account for slight variations in the amount of cells harvested from the agar plates. This experiment was performed on n = 3 independent experiments per strain.

### Assessment of phenylalanine biosynthesis

To determine if saPDT is required for phenylalanine biosynthesis, UAMS-1 (pMK4), *nos*::*erm* mutant (pMK4), Δ*pdt* mutant (pMK4), and corresponding complement strains were grown in chemically-defined media (CDM) [33] in the presence and absence of phenylalanine. In brief, each strain was cultured overnight in TSB containing 5 µg/ml Cm, followed by centrifugation to harvest cells. Cell pellets were each washed once in CDM lacking phenylalanine, then resuspended in the same media. These washed cell suspensions were then used to inoculate 1 ml of CDM with phenylalanine or CDM lacking phenylalanine to an OD_600_ = 0.02. Aliquots (200 µl) of each diluted culture were transferred in triplicate to wells of a 96-well tissue culture plate (Costar 3596), and grown at 37°C. OD_600_ readings were measured in a Synergy HT microplate reader (Biotek) every 2 hours over a 24 hour growth period. This experiment was performed on n = 4 biological replicates (acquired in 2 independent experiments) per strain.

### Fluorescent detection of endogenous NO/RNS

To detect intracellular accumulation of NO and RNS in *S. aureus*, a fluorescent assay using the cell-permeable stain 4-Amino-5-Methylamino-2',7'-Difluorofluorescein (DAF-FM) Diacetate (Invitrogen Life Technologies) was developed. To validate the use of this stain as a measure of intracellular NO/RNS in *S. aureus*, strain UAMS-1 was grown overnight in TSB supplemented with 3% (wt/vol) NaCl and 0.5% (wt/vol) glucose (TSB-NaGlc). Overnight cultures were then diluted to an OD_600_ = 0.05 in TSB-NaGlc, and 1 ml aliquots were transferred to wells of a 24-well tissue culture plate (Costar 3524; previously coated overnight at 4°C with 20% human plasma) and grown for 7 hours at 37°C. Following growth, the total well (biofilm + supernatant) was harvested, centrifuged and cell pellets were resuspended in 1× Hank's Buffered Salt Solution (HBSS) buffer containing 5 µM DAF-FM diacetate. Cell suspensions were incubated for 1 hour at 37°C, then cells were collected by centrifugation, washed once in HBSS buffer, and resuspended in HBSS alone (“untreated”) or HBSS supplemented with 100 µM Diethylamine (DEA; Sigma) or 100 µM DEA/NO (Cayman Chemicals). Aliquots (200 µl) of each untreated or treated cell suspension were immediately transferred in triplicate to wells of a Costar 3904 96-well plate, which was incubated at 37°C in a Biotek Synergy HT fluorescent plate reader. Fluorescence (EX/EM 485±10/516±10) and OD_600_ measurements were recorded after 30 minutes, and data were reported as relative fluorescent units (RFU) per OD_600_ of each well. Where indicated, 2-(4-carboxyphenyl)-4,5-dihydro-4,4,5,5-tetramethyl-1H-imidazolyl-1-oxy-3-oxide, monopotassium salt (cPTIO), an NO scavenger, was added to cell suspensions to a concentration of 150 µM during the 1 hour DAF-FM staining step.

To determine the relative levels of endogenous NO/RNS of *S. aureus* when grown on TSA plates, UAMS-1 (pMK4), *nos*::*erm* mutant (pMK4), *nos* complement (*nos*::*erm* mutant containing pMKnos) and *pdt* complement (*nos*::*erm* mutant containing pMKpdt) strains were cultured for 26 hours on TSA +5 µg/ml Cm. Cells were harvested from each plate by scraping with a sterile toothpick, and resuspended in 1 ml HBSS. Cell pellets were harvested by centrifugation, washed once in HBSS buffer, and then resuspended in HBSS containing 5 µM DAF-FM. Aliquots (200 µl) of cell suspension were immediately transferred in quadruplicate to wells of a Costar 3904 96-well plate, which was incubated at 37°C in a Synergy HT fluorescent plate reader. Fluorescence and OD_600_ measurements were recorded after 90 minutes, and data were reported as RFU per OD_600_ of each well.

### Hydrogen peroxide sensitivity assay

The sensitivity of UAMS-1 (pMK4), *nos*::*erm* mutant (pMK4), Δ*pdt* mutant (pMK4), and corresponding complement strains were assessed for sensitivity to H_2_O_2_ based on the method reported by Nudler et al. [15]. In brief, each strain was inoculated in LB broth (+ selective antibiotic, as needed), and grown overnight at 37°C and 250 RPM. These cultures were diluted to an OD_600_ = 0.05 in 20 ml LB (no antibiotic) in a 250 ml Erlenmeyer flask. After growth for 4.75–5 hours at 37°C and 250 RPM, 1 ml was removed for serial dilution, track plating [34] and CFU/ml determination, and the remaining culture was incubated in the presence of 250 mM H_2_O_2_ for 2 hours. After this 2 hour treatment, 1 ml of culture was removed from each flask for serial dilution and track plating to determine CFU/ml.

### Murine model of sepsis and dissemination

These experiments were conducted as described previously [35], [36]. Briefly, 6 week old, female CD-1 Swiss mice were purchased from Charles River Laboratories, and housed at the vivarium in the College of Medicine, University of South Florida. Animals were housed in standard metal cages (2 per cage), with *ad libitum* food and water access. For infection purposes, previously-stocked cultures of UAMS-1 and KR1010 were thawed, washed twice in PBS, and diluted in PBS to 1×10^9^ CFU/ml. Mice were randomly assorted into two groups (n = 9 mice per strain) and subsequently inoculated by tail vein injection with 100 µl bacterial suspension, giving a final inocula of 1×10^8^ CFU/ml. The infection was allowed to proceed for 7 days, or until mice reached a pre-moribund state (used as a measure of mortality). Mice were monitored every 3 hours for the first 24 hours after inoculation, then every 8 hours (up to 72 hours post-inoculation), and twice daily for the remaining infection period. The criteria used for determining the pre-moribund state included development of a hunched posture, labored breathing, decreased activity, immobility, inability to eat or drink, and ruffled fur. Mice were then euthanized with carbon dioxide, and death was verified by the cessation of cardiovascular and respiratory activity. The liver, kidneys, heart, and lungs were collected and stored at −80°C. Any mouse sacrificed before day 7 was recorded for mortality, and their organs were stored for enumerating bacterial burden. Each organ was subsequently homogenized in 3 ml sterile PBS, and the CFU/organ determined via serial dilution and viable count enumeration. This study was performed in strict accordance with the recommendations in the Guide for the Care and Use of Laboratory Animals of the National Institutes of Health. The protocol was approved by the Institutional Animal Care and Use Committee of the University of South Florida (Permit Number: A-4100-01).

### Statistical analyses

All statistical analyses used for the *in vivo* data were performed using SAS software (version 9.2, SAS Institute, Cary, NC). The distribution of data was determined in SAS through tests for normality (*SAS proc univariate*) and equality of variance (*SAS proc ttest*). The statistical significance of bacterial recovery from the murine model of sepsis was evaluated using a Mann-Whitney Test. Mortality was measured using a Log Rank and Chi-square test with 1-degree of freedom. For all *in vivo* statistical analyses the significance level was set at α = 0.05. *In vitro* assays were subjected to statistical analysis using Sigmaplot version 12.5 software (Build 12.5.0.38, Systat Software, Inc.). Data were tested for normality and equal variance, then analyzed with a parametric (if normality and/or equal variance tests passed) or non-parametric (if normality and/or equal variance tests failed) one-way ANOVA, followed by an appropriate multiple-comparison test to detect differences between individual groups.

## Results

### The *S. aureus nos* and *pdt* genes comprise a co-transcribed operon

**Figure 1:**
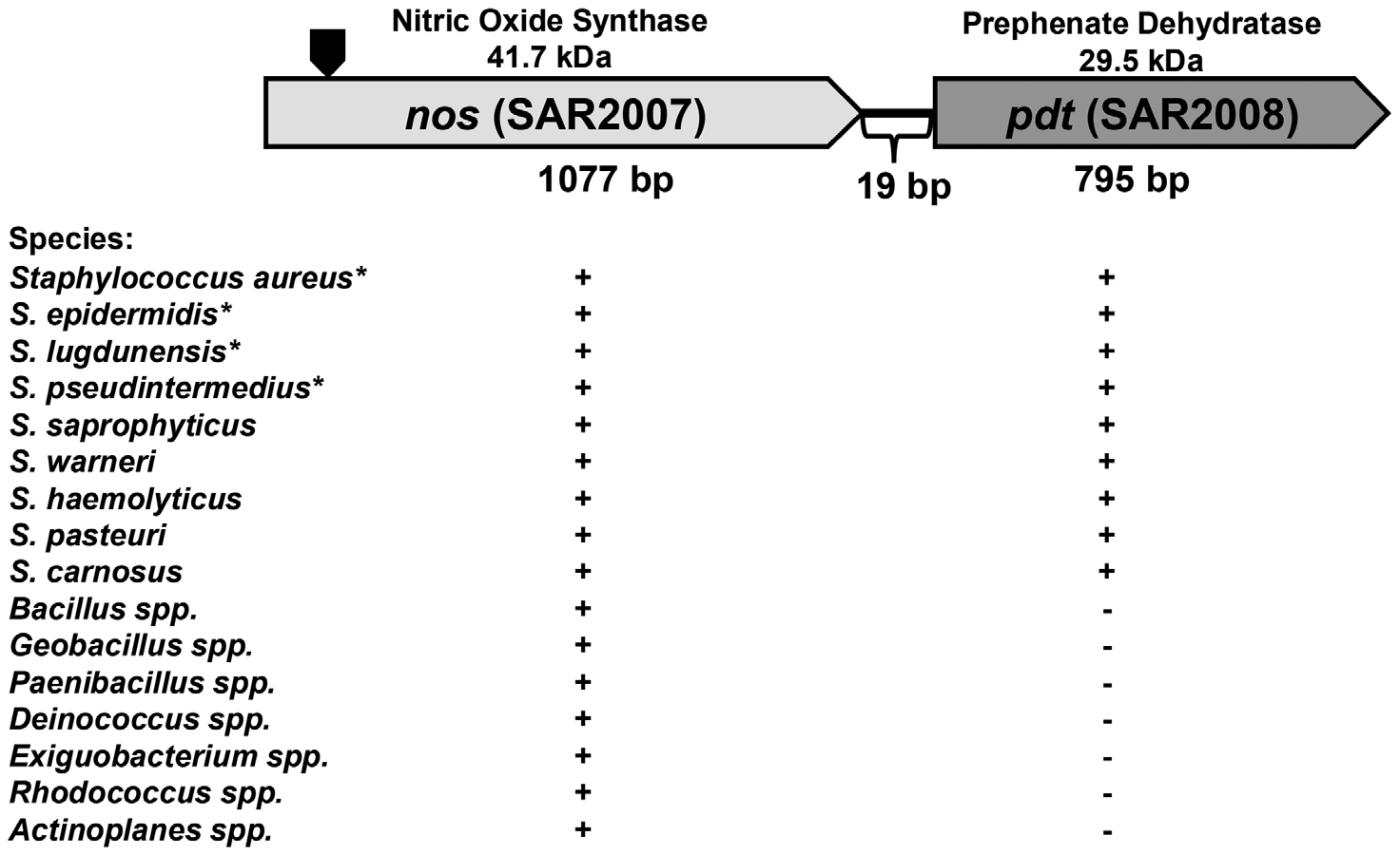
Genetic arrangement of the *S. aureus nos* and *pdt* genes. The representative bacterial genera and/or species that contain a *nos* gene are listed below the *S. aureus nos-pdt* operon diagram. The “+” designation under *nos* indicates its presence in each respective genome, whereas the “+/−” designation under *pdt* indicates if this gene is located immediately downstream of *nos* in each respective bacterial genera/species. The predicted molecular weight (kDa) of the *nos* and *pdt* ORFs are indicated above each gene, and the size (bp) of each gene is shown below. Gene designations refer to the MRSA252 genome. The site where the *erm* resistance cassette was inserted to create the *nos*::*erm* mutant (KR1010) is indicated by the black block arrow. *denotes that all sequenced strains in NCBI contain the indicated genes.

Inspection of the *S. aureus nos* genomic region revealed the presence of an ORF located only 19-bp downstream of the *nos* gene. This ORF (SAR2008 of the MRSA252 genome, herein referred to as *pdt*) encodes a predicted prephenate dehydratase (saPDT) enzyme, which catalyzes the formation of phenylpyruvate from prephenate during phenylalanine biosynthesis [23]. A conserved domain search [37] of the *S. aureus pdt* ORF revealed the presence of signature catalytic (PDT; IPR001086) and regulatory (ACT; IPR002912) domains present in mono-functional PDT enzymes that are common to Gram-positive bacteria [23]. A BLAST search [38] using the *S. aureus* MRSA252 nucleotide sequence spanning the *nos* and *pdt* ORFs revealed that this organization is present in all sequenced *S. aureus* genomes, as well as all sequenced coagulase-negative staphylococcal genomes currently available in the National Center for Biotechnology Information (NCBI) genome database (Fig. 1). As reported previously [3] and verified by our own BLAST search of all sequenced microbial genomes in the NCBI database, numerous other bacterial genomes contain a gene encoding a predicted NO-synthase that is highly homologous to the *S. aureus nos* ORF (>50% amino acid identity). However, inspection of the genomic context of *nos* in these other bacteria revealed that the putative *nos-pdt* operon structure appears to be unique to the staphylococci (Fig. 1).

**Figure 2:**
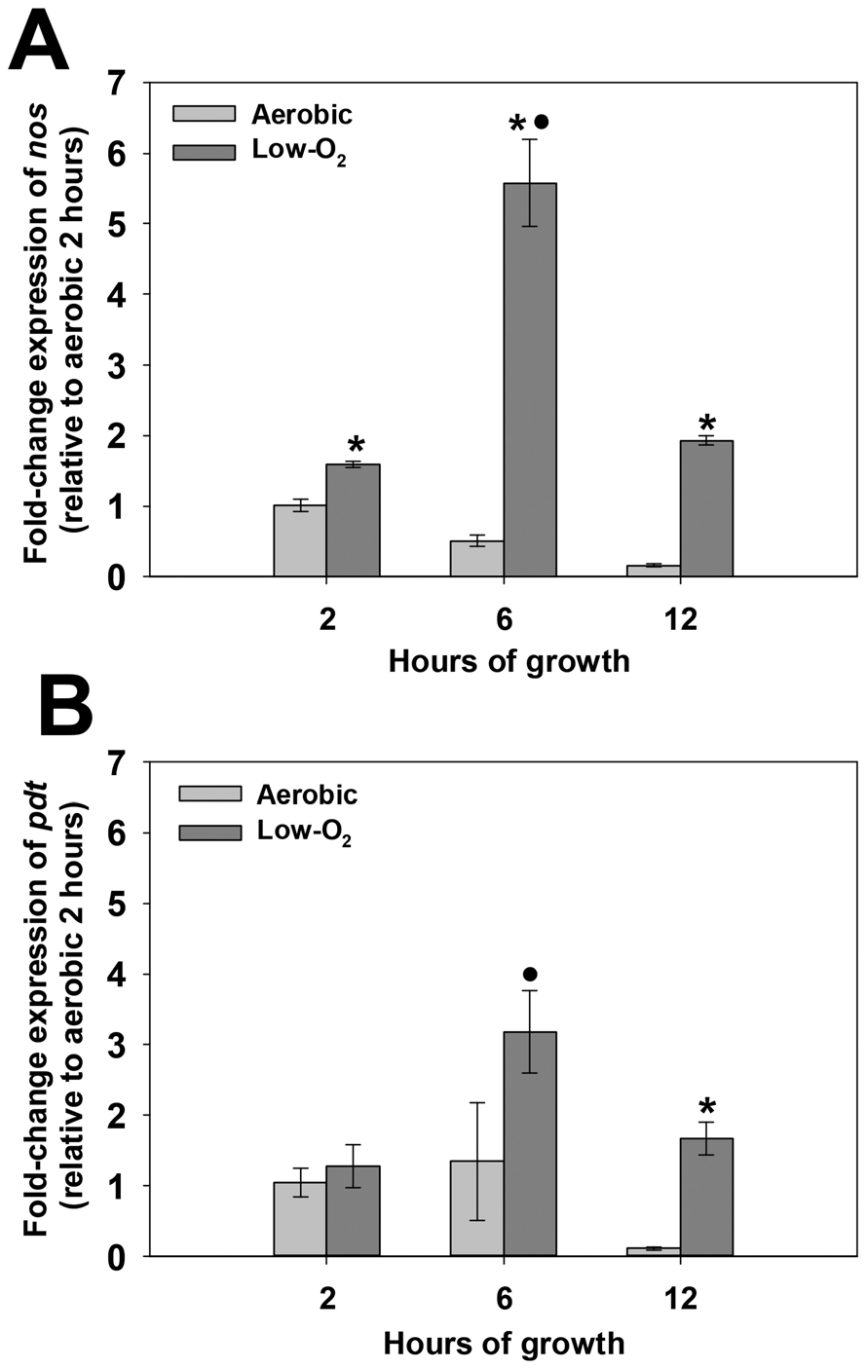
Growth-phase dependent *nos* (A) and *pdt* (B) expression. Total RNA was isolated at the indicated time points from n = 3 biological replicates of UAMS-1 grown under highly aerated (light grey bars; 250 RPM, 1∶10 volume-to-flask ratio) or low-oxygen (dark grey bars; 0 RPM, 7∶10 volume-to-flask ratio) conditions. Quantitative real-time PCR was performed on reverse-transcribed cDNA from each sample using *nos* (A) or *pdt* (B) specific primers. The Livak (2^-ΔΔCt^) method was used to determine relative fold change of *nos* and *pdt* expression, using measured *sigA* expression as the reference gene and the 2 hour aerobic sample as the calibrator. Error bars  =  standard error of the mean (SEM). *denotes statistical significance between aerobic and low-oxygen fold-change values at each indicated time point (Two-Tailed T-Test, p<0.01); •denotes statistical significance relative to t = 2 hour low-oxygen time point (Dunnett's Test, p<0.05).

**Figure 3:**
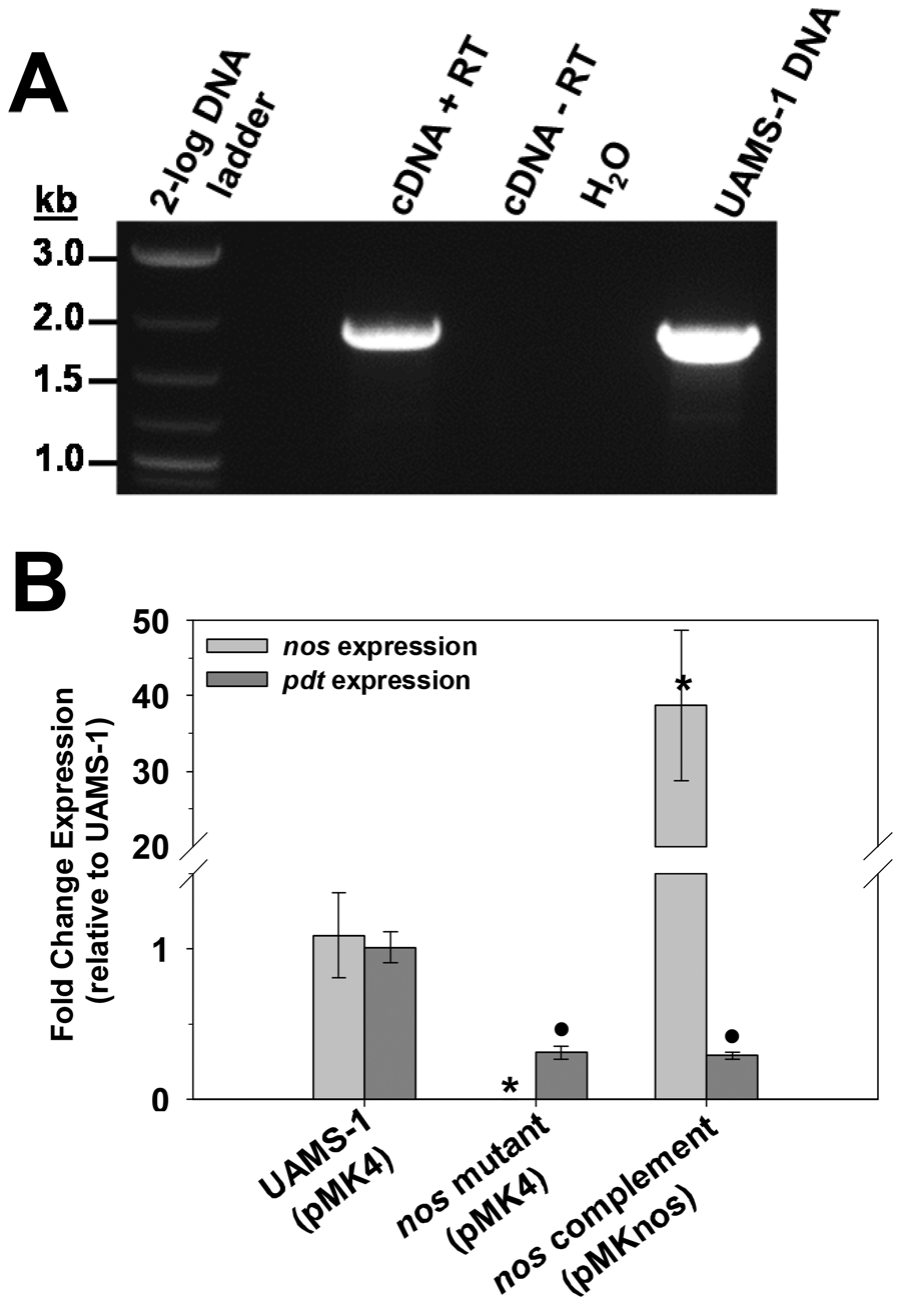
Co-transcription of *nos* and *pdt* genes. **A:** 0.750 µg of UAMS-1 RNA isolated from 6-hour low-oxygen cultures was converted to cDNA using the iScript select cDNA kit (BioRad) and primer pdt5-R. As a control, these reactions were also performed in the absence of reverse-transcriptase (RT) enzyme, to check for genomic DNA contamination of the RNA. Equal amounts of RT+ and RT- cDNA samples, as well as UAMS-1 genomic DNA and no template control, were used as templates in a PCR reaction containing the primer pair nos5-F/pdt5-R, which amplifies a product spanning the region between the *nos* start codon and *pdt* stop codon. Data are representative of results obtained from RNA isolated from n = 3 biological replicates. **B:** Total RNA was isolated from n = 3 biological replicates each of UAMS-1 (pMK4; wild-type), KR1010 (pMK4; *nos* mutant), and KR1010 (pMKnos; *nos* complement) grown at 37°C for 6 hours under low-oxygen (0 RPM, 7∶10 volume-to-flask ratio) conditions. Quantitative real-time PCR was performed on reverse-transcribed cDNA from each sample using *nos* and *pdt*-specific primers. The Livak (2^-ΔΔCt^) method was used to determine relative fold change of expression of each gene, using measured *sigA* expression as the reference gene and the 2 hour aerobic sample as the calibrator. Error bars  =  SEM. *denotes statistical significance of *nos* expression relative UAMS-1 (Student-Newman-Keuls Test, p<0.05); •denotes statistical significance of *pdt* expression relative UAMS-1 (Student-Newman-Keuls Test, p<0.05).

To gain insight into the growth conditions under which *nos* and *pdt* are expressed, quantitative real-time PCR (qPCR) was performed on RNA isolated from aerobic and low-oxygen cultures at early-exponential (2 hours growth), late exponential (6 hours growth) and stationary (12 hours growth) growth phase (Fig. 2). The expression patterns of *nos* and *pdt* were similar, in that RNA levels of each gene were greatly increased under low-oxygen growth compared to aerobic growth. Furthermore, expression of both of these genes was growth-phase dependent, peaking at late exponential growth phase, and declining in stationary phase. Co-transcription of *nos* and *pdt* was verified by synthesizing cDNA from UAMS-1 RNA using a primer that anneals to the 3' end of the *pdt* gene, followed by PCR using primers designed to amplify a 1.9 kb product that spans the start codon of *nos* and stop codon of *pdt*. As shown in Fig. 3A, a distinct 1.9 kb PCR product was amplified using cDNA as template, confirming that *nos* and *pdt* are indeed co-transcribed. This amplification only occurred when reverse-transcriptase (RT) enzyme was present in the cDNA synthesis reaction, indicating that this product was not amplified from contaminating genomic DNA. Because the *nos* mutant used in this study was created by inserting an erythromycin resistance gene cassette into the *nos* coding sequence, the effect on *pdt* transcription in this mutant was also assessed using quantitative real-time PCR (Fig. 3B). Comparison of *pdt* expression in the *nos* mutant by this method revealed that *pdt* RNA levels were about 3-fold less than the wild-type strain in low-oxygen 6 hour cultures, and *pdt* expression was not restored when the *nos* mutant was complemented with *nos* expression from a plasmid. These results collectively demonstrate that the *nos* and *pdt* genes represent a co-transcribed operon, and that the insertion mutagenesis used to create the *nos* mutant has a partial polar effect on *pdt* expression when cultured in TSB.

### *S. aureus* PDT is required for growth in the absence of phenylalanine but does not affect oxidative stress resistance and carotenoid pigmentation

**Figure 4:**
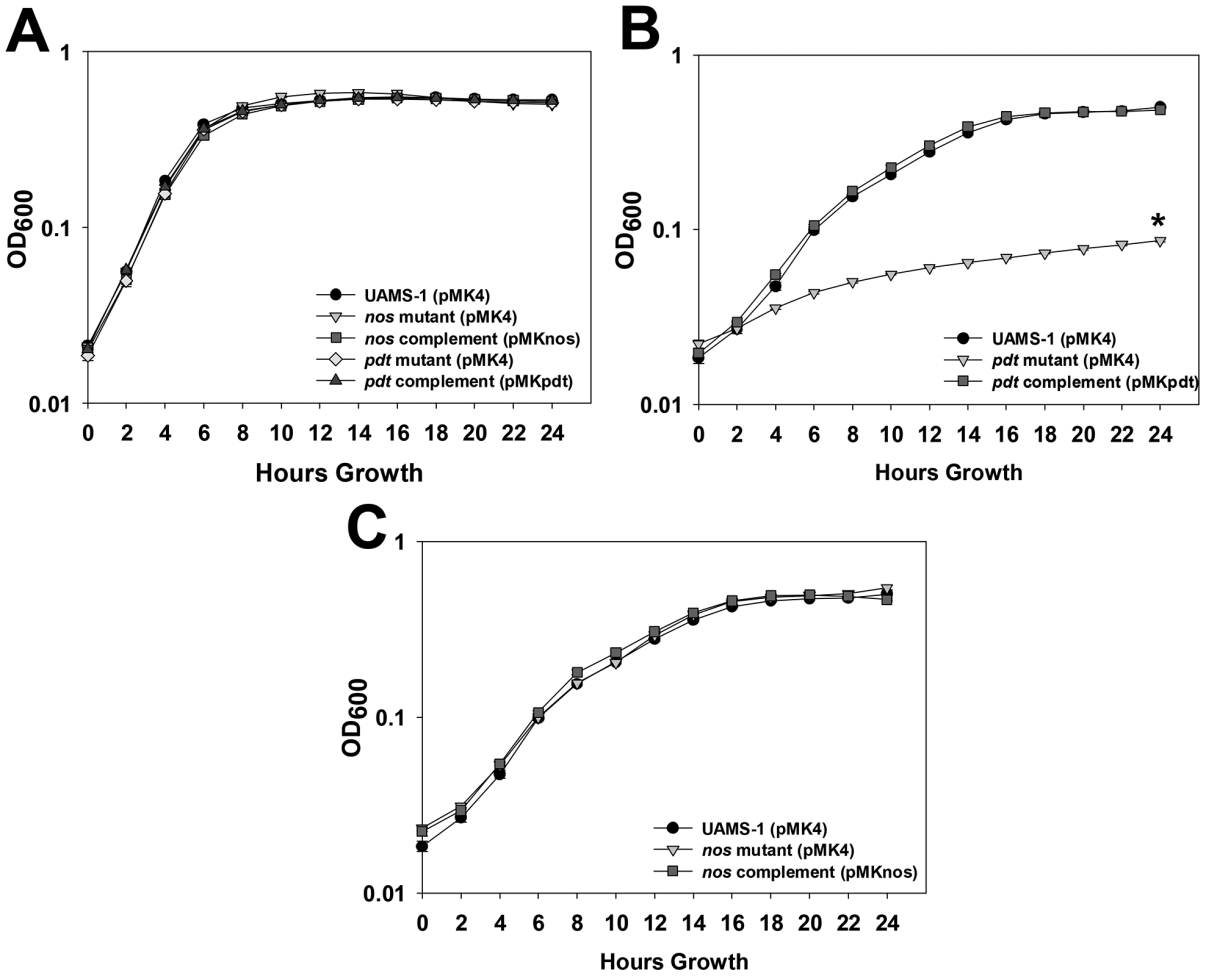
Effects of *nos* and *pdt* on growth in chemically-defined media. UAMS-1 (pMK4; wild-type), KR1010 (pMK4; *nos* mutant), KR1010 (pMKnos; *nos* complement), KR1013 (pMK4; *pdt* mutant), and KR1013 (pMKpdt; *pdt* complement) were grown in chemically-defined media in the presence (A) or absence (B and C) of phenylalanine. Cultures were inoculated to an OD_600_ = 0.02, and grown statically in a 96-well plate for 24 hours at 37°C. OD_600_ readings were measured every 2 hours using a microplate reader. Data represents the average of n = 4 biological replicates acquired in 2 independent experiments. Error bars  =  SEM. *statistical significance of *pdt* mutant (pMK4) OD_600_ compared to UAMS-1 (pMK4) at t = 24 hours growth (p<0.05, Tukey Test).

The crystal structure of saPDT has been previously solved and the predicted enzymatic function of this enzyme (conversion of phenylpyruvate to prephenate) has been confirmed *in vitro* [23]. However, the operon structure of *nos* and *pdt* suggest that the functions of each encoded enzyme may be inter-related. Therefore, the ability of wild-type, *nos* mutant, *pdt* mutant, and corresponding complement strains to grow in the presence and absence of phenylalanine was compared using a chemically-defined media (CDM; Fig. 4). In complete CDM (containing phenylalanine), all wild-type, mutant, and complement strains displayed similar growth patterns over a 24-hour period, and each achieved a similar OD_600_ after 24 hours growth (Fig. 4A). However, in CDM lacking phenylalanine, the *pdt* mutant displayed greatly reduced growth relative to the wild-type strain, and reached an OD_600_ that was 83% less than the wild-type strain after 24 hours (Fig. 4B). This growth defect was fully complemented by expressing the *pdt* gene in trans on a plasmid in the *pdt* mutant strain, confirming a requirement for this enzyme in *S. aureus* phenylalanine biosynthesis. The pattern of *nos* mutant growth was nearly identical to the wild-type strain in the absence of phenylalanine (Fig. 4C), even though this mutation had a partial polar effect on *pdt* expression when grown in TSB (Fig. 3B). Data retrieved from a previously-published RNA-seq experiment performed on *S. aureus* strain USA300 HOU [39] suggests that there may be other potential promoter sites within the *nos*-*pdt* operon capable of driving *pdt* expression (Fig. S1 in File S1). At least two of these regions are located downstream of the inserted *erm* cassette in the *nos* mutant, and may represent a potential reason why growth in the absence of phenylalanine was not impaired in the *nos* mutant. Overall these results suggest that (1) saNOS is not required for growth in the absence of phenylalanine, and (2) there is adequate expression of *pdt* in the *nos* mutant to support phenylalanine biosynthesis when grown in chemically-defined media.

**Figure 5:**
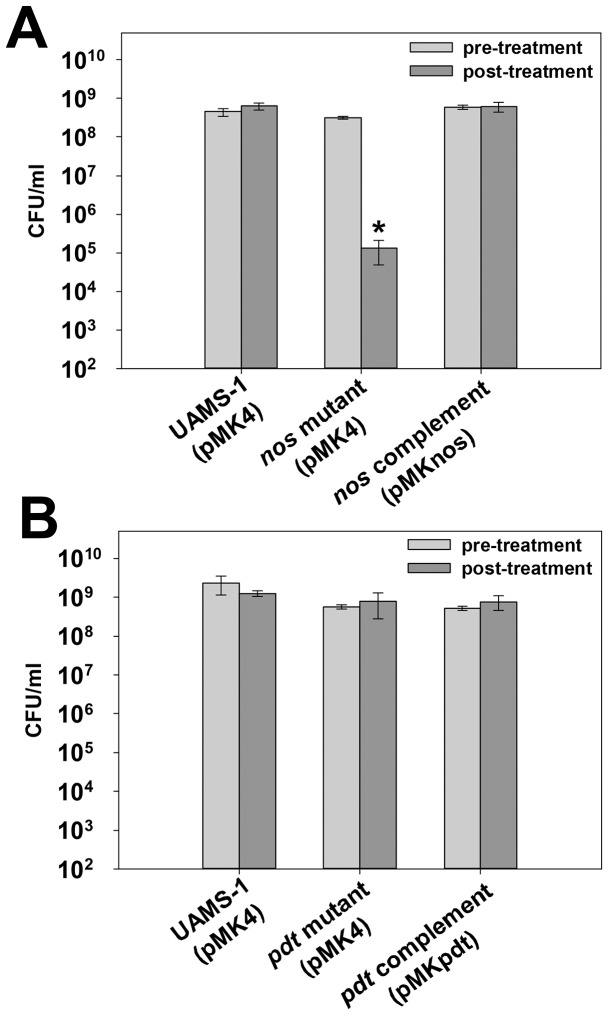
Effect of *nos* and *pdt* on oxidative stress resistance. **A:** UAMS-1 (pMK4; wild-type), KR1010 (pMK4; *nos* mutant) and KR1010 (pMKnos; *nos* complement) were cultured aerobically for 4.75–5 hours (mid-exponential growth phase) in LB media (“pre-treatment” time point, light grey bars) prior to addition of 250 mM H_2_O_2_. Cultures were then grown for 2 more hours to assess the effect of H_2_O_2_ treatment cell viability by CFU plating (“post-treatment” time point, dark grey bars). **B:** UAMS-1 (pMK4; wild-type), KR1013 (pMK4; *pdt* mutant), and KR1013 (pMKpdt; *pdt* complement) were analyzed for resistance to 250 mM H_2_O_2_ as described in panel A. All data represent the average of n = 5 independent experiments. Error bars  =  SEM. *statistical significance compared to UAMS-1 (pMK4) (p<0.05, Student-Newman-Keuls Test).

A role for saNOS in resistance to oxidative stress has been established [18], [20], [21], but a similar role for saPDT has not been previously explored. Given that *nos* [40] and *pdt* [40], [41] expression were shown by others to be induced in response to H_2_O_2_, it is possible that saPDT may also contribute to oxidative stress resistance, possibly by generating the α-keto acid anion phenylpyruvate that has been proposed to act as an antioxidant by direct chemical interaction with H_2_O_2_ [42]. To explore this possibility, wild-type, *nos* mutant, *pdt* mutant, and corresponding complement cultures were grown to late-exponential phase, and challenged with 250 mM H_2_O_2_ (Fig. 5). As expected, the *nos* mutant displayed a significant (>3-log) reduction in cell viability after H_2_O_2_ challenge compared to the wild-type strain, and this phenotype was fully-restored by supplying the *nos* gene on a plasmid (Fig. 5A). However, the sensitivity of the *pdt* mutant to oxidative stress was unaffected in this assay (Fig. 5B), suggesting that, at least in this *in vitro* growth condition, saPDT does not appear to function in an antioxidant capacity.

**Figure 6:**
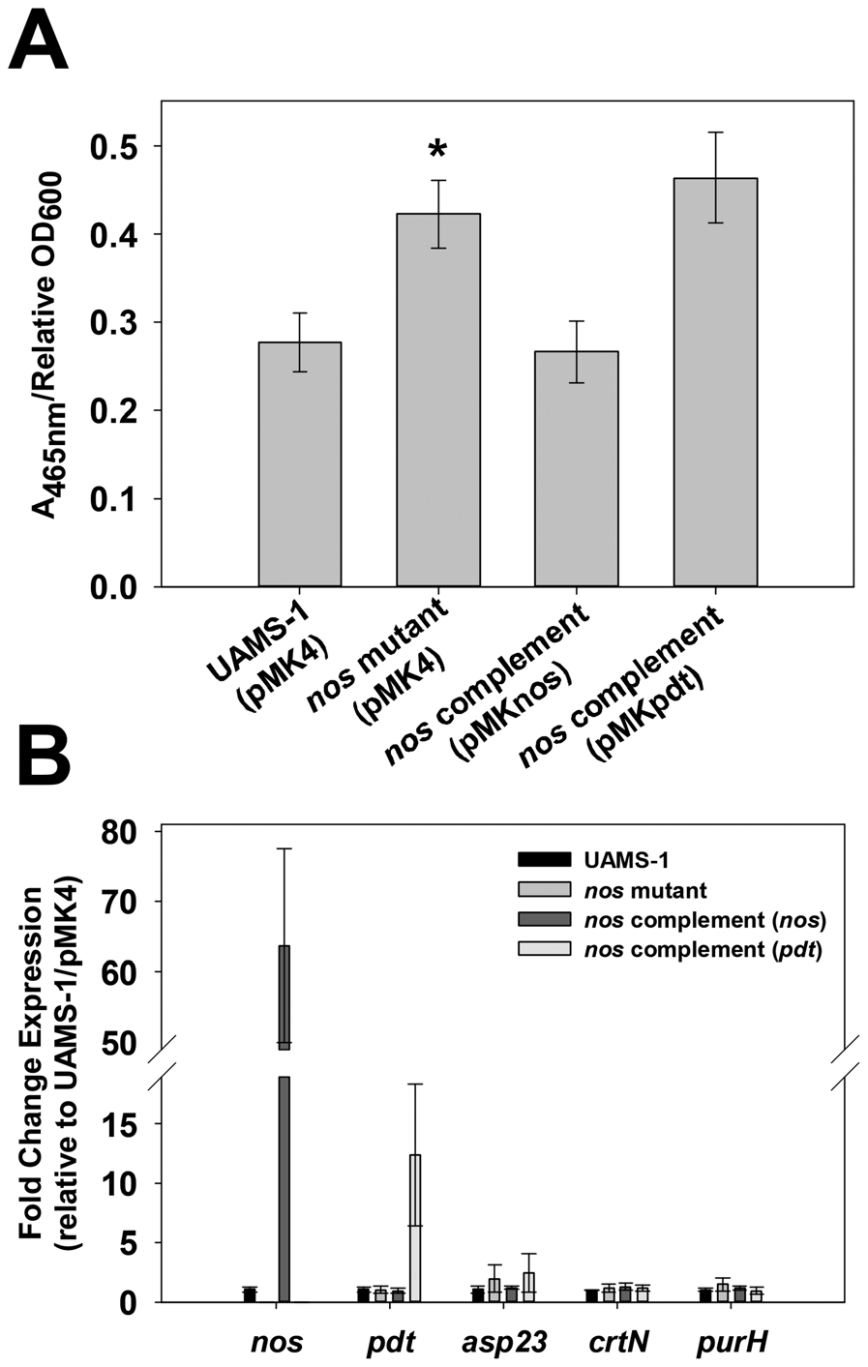
Effect of *S. aureus nos* mutation on carotenoid pigmentation. **A:** UAMS-1 (pMK4; wild-type), KR1010 (pMK4; *nos* mutant), KR1010 (pMKnos; *nos* complement), and KR1010 (pMKpdt; *nos* mutant containing *pdt* complement plasmid) were grown for 48 hours at 37°C on TSA plates. Pigments from each culture were extracted with methanol, the A_465 nm_ of each sample was measured, and standardized by dividing by the sample's corresponding relative OD_600_ value (measured prior to methanol extraction step). Results represent the average of n = 3 independent experiments, error bars  =  SEM. *statistical significance compared to UAMS-1 (pMK4) (p<0.05, Student-Newman-Keuls Test). **B:** Total RNA from 28 hour TSA plate cultures of UAMS-1 (pMK4), *nos*::*erm* mutant (pMK4), *nos* complement and *pdt* complement strains (n = 3 biological replicates each) was reversed-transcribed to cDNA, and subjected to quantitative real-time PCR using primers specific for detecting *nos*, *pdt*, *asp23*, *crtN*, and *purH* expression. The Livak (2^-ΔΔCt^) method was used to determine relative fold change expression of each gene, using measured *sigA* expression as the reference gene and the UAMS-1 (pMK4) sample as the calibrator.

During routine culturing of the wild-type, *nos* mutant, *pdt* mutant, and complement strains used in these studies, the *nos* mutant consistently appeared to display increased carotenoid (golden-yellow) pigmentation when cultured on TSA agar plates. This observation was verified by measuring the A_465 nm_ of methanol-extracted carotenoid pigments from 48-hour TSA plate cultures of wild-type, *nos* mutant, and complement strains (Fig. 6A). Consistent with qualitative observations, the *nos* mutant displayed a statistically-significant (p<0.05, Student-Newman-Keuls Test) increase in pigmentation compared to the wild-type strain. Furthermore, pigment was restored to wild-type levels by complementation with a *nos*-expressing plasmid but not with a *pdt*-expressing plasmid, verifying that increased pigmentation of the *nos* mutant is solely due to loss of saNOS function. To determine if increased pigmentation in the *nos* mutant was a result of altered expression of pigment-related genes, qPCR was employed to measure changes in expression of *crtN* (a member of the carotenoid biosynthesis operon [43]), *asp23* (alkaline shock protein 23, highly-regulated by alternative sigma factor B [44], [45]), and *purH* (purine biosynthesis gene H), as mutation in these genes and/or regulatory circuits have each been shown to greatly alter pigment production [43], [45], [46]. Surprisingly, *asp23*, *crtN*, and *purH* RNA levels were not significantly different between wild-type, *nos* mutant, and *nos* complement strains (Fig. 6B), suggesting that increased pigmentation of the *nos* mutant is not due to altered expression of these genes.

### Detection of intracellular NO/RNS in *S. aureus* by DAF-FM diacetate

**Figure 7:**
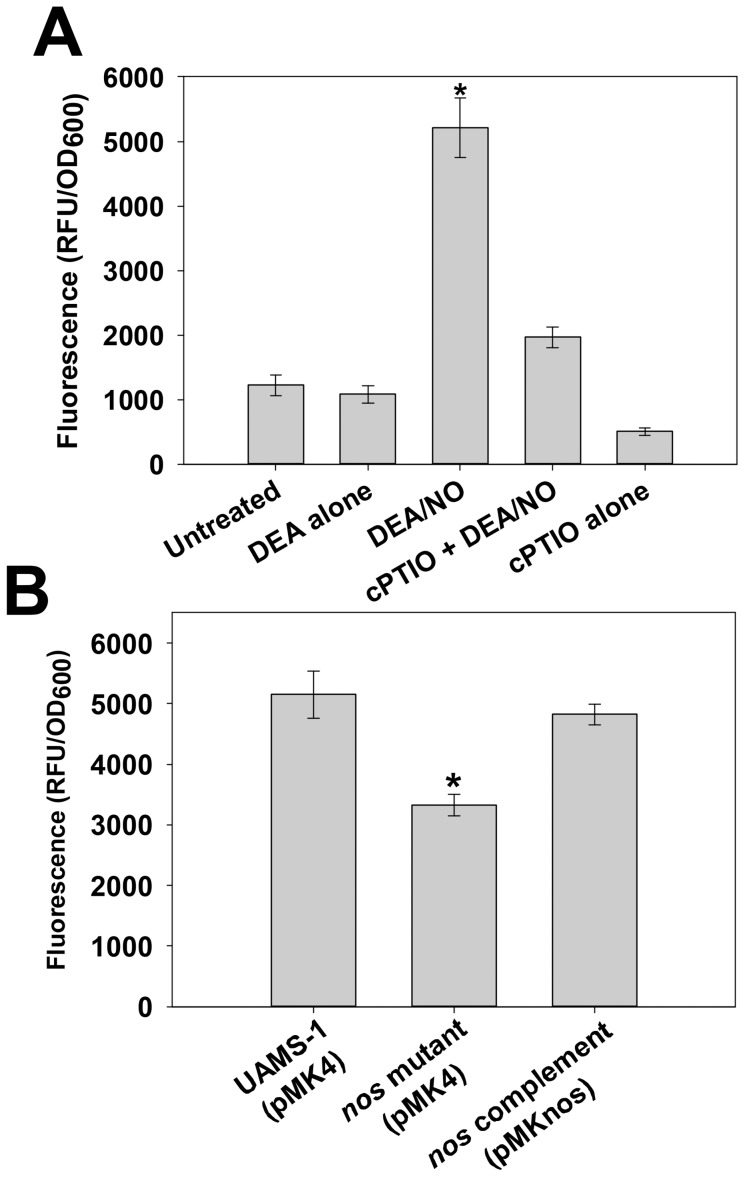
Detection of intracellular NO/RNS with DAF-FM diacetate. **A:** Cells harvested from replicate UAMS-1 7 hour static biofilms were resuspended in HBSS containing 5 µM DAF-FM diacetate. After incubation for 1 hour at 37°C, cells were collected by centrifugation, washed, and resuspended in HBSS alone (“untreated”) or HBSS supplemented with 100 µM DEA or 100 µM DEA/NO. Where indicated, 150 µM cPTIO (NO scavenger) was added to cell suspensions during the 1 hour DAF-FM diacetate staining step. Aliquots (200 µl) of each cell suspension were immediately transferred to a 96-well plate, and incubated at 37°C in a Synergy HT fluorescent plate reader. Fluorescence and OD_600_ measurements were recorded after 30 minutes, and data were reported as relative fluorescent units (RFU) per OD_600_ of each well. Data represents the average of n = 3 independent experiments, error bars  =  SEM. *statistical significance compared to untreated UAMS-1 (p<0.05, Tukey Test). **B:** UAMS-1 (pMK4; wild-type), KR1010 (pMK4; *nos* mutant), and KR1010 (pMKnos; *nos* complement) were grown on agar plates for 26 hours, followed by harvesting and resuspension in HBSS +5 µM DAF-FM dicaetate. Fluorescence (RFU) and OD_600_ readings were recorded after 90 minutes incubation in a microplate reader as described above. Data represents the average of n = 3 independent experiments, error bars  =  SEM. *statistical significance compared to UAMS-1 (pMK4) (p<0.05, Tukey Test).

To detect and compare relative levels of intracellular NO in *S. aureus*, an assay using the cell-permeable fluorescent stain DAF-FM diacetate was developed. This compound diffuses into cells and once inside, is cleaved by intracellular esterases to liberate weakly-fluorescent DAF-FM, which in turn becomes highly fluorescent upon reaction with NO or other specific RNS [47]. To validate the use of this stain as an indirect indicator of intracellular NO levels in *S. aureus*, wild-type biofilms grown for 7 hours were subjected to DAF-FM diacetate staining, followed by treatment with HBSS buffer alone, DEA/NO (a short half-life NO donor), DEA alone, cPTIO (an NO scavenger), and DEA/NO + cPTIO (Figure 7A). As expected, treatment with DEA/NO resulted in a marked increase in DAF-FM fluorescence in *S. aureus* cells, whereas treatment with DEA alone did not affect fluorescence relative to the untreated condition. Interestingly, treatment of cells with cPTIO alone decreased intracellular DAF-FM fluorescence relative to untreated cells (buffer alone), suggesting that this NO scavenger was able to reduce the level of endogenous NO detectable by intracellular DAF-FM. Furthermore, when cPTIO-treated cells were subsequently exposed to NO donor, the fluorescent signal did not greatly increase compared to the untreated sample. Collectively, these results validate the use of DAF-FM diacetate as a fluorescent tool to indirectly measure intracellular NO levels in *S. aureus*.

DAF-FM diaceate was initially used to measure relative endogenous NO levels in late-exponential phase low-oxygen TSB cultures of the wild-type, *nos* mutant, and complement strains, as *nos* expression was increased at this phase of low-oxygen growth (Fig. 2). However, no detectable differences in DAF-FM fluorescence were observed (Fig. S2 in File S1), suggesting that saNOS activity may be minimal or very transient at this phase of growth. Alternatively, its contribution to endogenous NO in this growth condition may be masked relative to other cellular sources of NO/RNS. Since phenotypic changes in pigmentation were observed when these strains were cultured on TSA plates (Fig. 6A), 28-hour TSA plate cultures of wild-type, *nos* mutant, and complement strains were also assessed for endogenous NO levels by staining with DAF-FM diacetate (Fig. 7B). Intriguingly, DAF-FM fluorescence was significantly (p<0.05, Tukey test) decreased relative to the wild-type and complement strains, suggesting that saNOS enzyme activity and/or expression is increased in *S. aureus* when cultured on TSA plates. Given that the level of DAF-FM fluorescence only decreased by about 35% in the *nos* mutant, it is likely that other cellular sources of endogenous NO and/or RNS are also present that are able to react with DAF-FM in this assay.

### Contribution of saNOS to survival in a murine sepsis model

**Figure 8:**
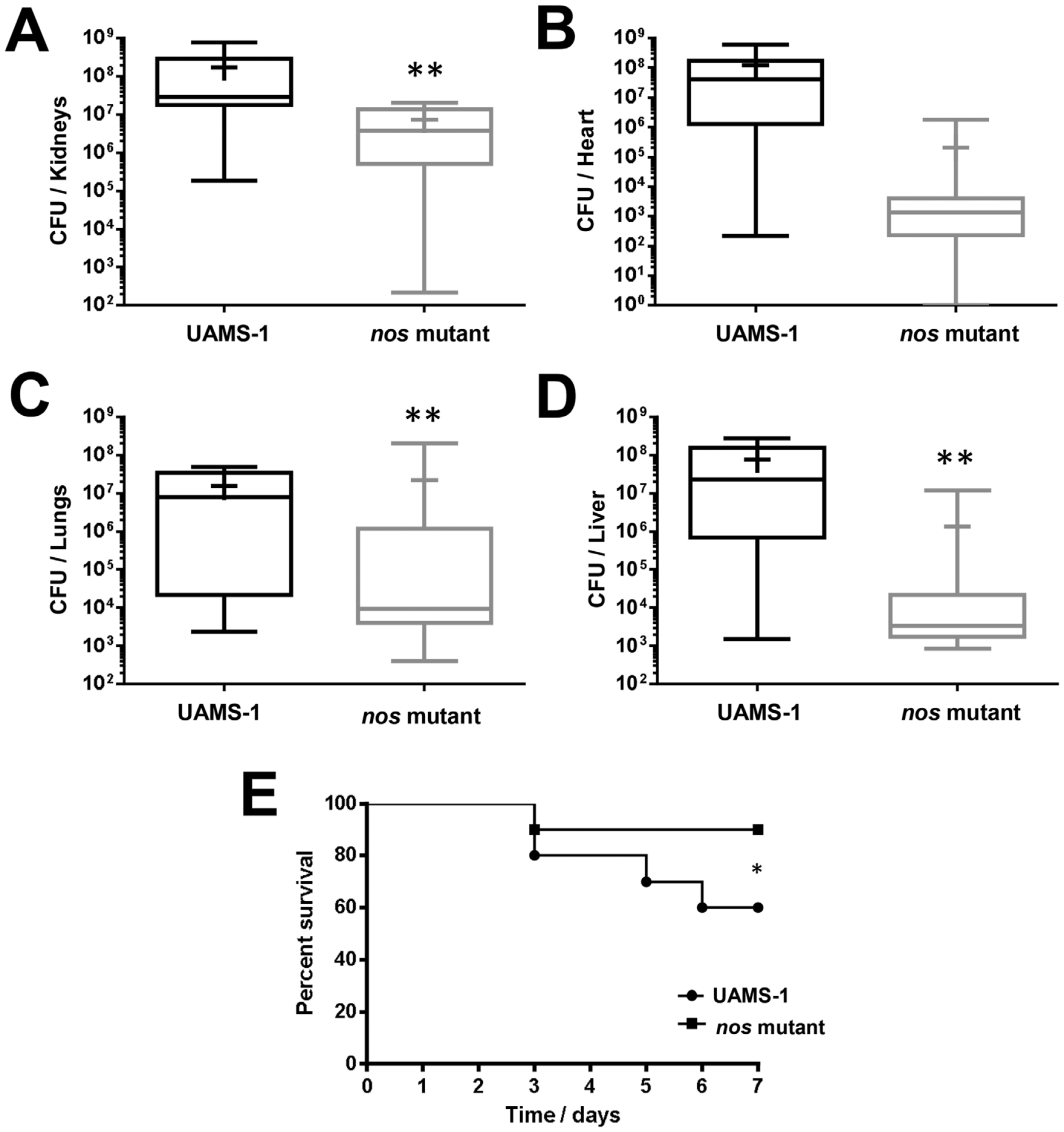
Effect of *nos* on *S. aureus* virulence. 1×10^8^ CFU/ml of UAMS-1 (wild-type) and KR1010 (*nos* mutant) cultures were each injected into the tail veins of female CD-1 Swiss mice (n = 9 mice per group). The infection proceeded for 7 days, or until mice reached a pre-moribund state (used as a measure of mortality). Mice were then euthanized and organs were collected and stored at −80°C prior to homogenization and determination of recovered CFU/organ. Graphs represent the calculated bacterial burdens for the kidneys (A), heart (B), lungs (C) and liver (D), and the percent survival (E). For A–D, data are graphed as box-whisker plots, indicating the 25^th^, median, and 75^th^ percentiles (box lines), the 90^th^ and 10^th^ percentiles (error bars), and mean (+) of each data-set. *denotes statistically significant difference of mortality (p<0.05, Log Rank and Chi Squared Test with 1-degree of freedom), **denotes statistical significance of bacterial recovery (p<0.05, Mann-Whitney Test).

Although the contribution of saNOS to MRSA virulence has been previously-demonstrated in a murine abscess model of infection [21], a role for this enzyme in virulence of MSSA has not been reported. Therefore, the virulence of MSSA strain UAMS-1 and its isogenic *nos* mutant was compared using a previously-published murine sepsis model of infection [35], [36]. As demonstrated in Figs. 8A–8D, the bacterial load (as measured by CFU/organ) in the lungs, kidneys, and liver were significantly (p<0.05, Mann-Whitney Test) lower in the *nos* mutant-infected mice compared to wild-type infected mice. Although not a statistically significant difference, bacterial counts from the heart were also decreased in *nos* mutant-infected mice relative to the wild-type infected mice. As well, the *nos* mutant-infected mice displayed significantly (p<0.05, Log Rank and Chi Squared Test with 1-degree of freedom) less mortality than the wild-type infected mice in this model of infection, with 8/9 *nos* mutant-infected mice and 6/9 wild-type-infected mice surviving throughout the 7-day infection (Fig. 8E). Given that a partial polar effect on *pdt* expression was observed in the *nos* mutant when grown in low-oxygen TSB cultures (Fig. 3B), it is possible that the *in vivo* effects of the *nos* mutant observed in this study are due, in part, to partial loss of *pdt* expression. These results, combined with previously-published data showing that a MRSA *nos* deletion mutant displayed decreased virulence in an abscess infection model [21], suggest that saNOS and/or saPDT contribute to *S. aureus* dissemination and survival *in vivo*.

## Discussion

An emerging body of evidence has demonstrated that bacterial NOS enzymes act as important regulators of cellular stress resistance and physiology. For example, NO-derived from the NOS of plant-pathogenic *Streptomyces* species is required for the nitration of thaxtomin phytotoxins, a modification that is essential for its phytotoxicity [12]. The NOS enzymes of *B. subtilis, B. anthracis*, and *S. aureus* have also been shown to promote resistance to oxidative stress imposed by hydrogen peroxide [14], [15], [21] and by certain antibiotics [18], [21]. This oxidative stress resistance has been proposed to be mediated by several mechanisms, including transient suppression of the enzymatic reduction of intracellular cysteines by NO (and subsequent inhibition of the Fenton reaction) [14], [15], NO activation of catalase activity [14], [15], and NO-induced expression and/or increased activity of superoxide dismutase (SOD) [18], [21]. Our current results have corroborated the contribution of saNOS to hydrogen peroxide resistance in the MSSA strain UAMS-1, and furthermore, have also illustrated that carotenoid pigmentation is increased in a *nos* mutant when grown on TSA plates (Fig. 6A), a phenotype that has not been previously described for *S. aureus nos* mutants. Staphyloxanthin, the major carotenoid pigment synthesized by a series of enzymes encoded by the *crt* operon [43], has been shown to be a potent antioxidant defense against hydrogen peroxide and the immune cell respiratory burst [48], [49], as well as conferring tolerance to host antimicrobial peptides [50] and fatty acids [51] by altering membrane fluidity. Therefore, at first glance, it appears paradoxical that a *S. aureus nos* mutant would be more sensitive to oxidative stress while displaying increased carotenoid pigmentation. However, the hydrogen peroxide challenge assay utilized in this study was performed during mid-exponential growth phase in planktonic cultures, a growth condition under which differences in staphyloxanthin production is unlikely to be observed, since maximal expression of *crtM* has been shown to occur in stationary phase in planktonic culture [46]. Although loss of saNOS does not appear to alter expression of the *crt* operon and regulators of *crt* expression when grown on TSA plates (Fig. 6B), it is possible that NO derived from saNOS may regulate the activity of one or more of the enzymes involved in the synthesis of staphyloxanthin or one of its precursors. Another possibility stems from the fact that the degree of conjugation (increased number of double bonds) in carotenoids tends to correlate to the observed intensity of orange-yellow pigmentation [52]. Since NO is known to have an affinity for biological membranes [53] and can attack the double bonds of β-carotene [54], it is possible that increased pigmentation observed in the *S. aureus nos* mutant is simply a result of loss of NO accumulation in the cell membrane.

In these presented studies, we have also optimized the use of DAF-FM diacetate to monitor relative intracellular NO levels in *S. aureus*. Using this technique, a decrease in intracellular NO/RNS was observed in the *S. aureus nos* mutant when cultured on TSA plates, a growth condition where increased pigmentation of the *nos* mutant was also observed. Although DAF-FM can also react with certain RNS such as nitrosonium ions, the majority of the fluorescent signal has been shown by others to be due to NO [55], [56]. Since the NO radical itself is relatively unstable and may quickly yield other RNS upon exposure to cellular components, intracellular DAF-FM fluorescence should be considered an indirect measurement of NO levels. In previous studies of *S. aureus* [21], *B. anthracis* [14], and *B. subtilis* [57] NOS activity, a NO-specific copper fluorescein (CuFL) stain [58], [59] was utilized to detect intracellular NO production in wild-type and *nos* mutants. When used in *S. aureus*, the cells were incubated in a buffer containing arginine prior to CuFL staining [21] and it was not specified in this study whether excess arginine was necessary to detect NO production. Arginine addition may artificially induce *nos* expression and/or enzyme activity in cells not normally producing *nos*, and may overestimate the actual number of cells that contain high-level saNOS enzyme activity when growing *in vivo*. Furthermore, CuFL specifically interacts with NO to yield high-level fluorescence, but it may be more difficult to detect endogenous NO if it is produced in a short biological window or if it reacts quickly with other cellular targets to yield other RNS. In these cases the use of DAF-FM diacetate may provide a viable alternative method of intracellular fluorescent NO detection.

The protective effects of endogenous NO observed in bacteria *in vitro* likely translate to an important contribution of NOS to the virulence of these Gram-positive pathogens during human infection. For example, a *B. anthracis nos* mutant displayed decreased survival when engulfed by macrophages, and an increased lethal dose (LD_50_) of spores compared to the parental strain in a murine model of systemic infection [14]. Furthermore, a *nos* mutant generated in a highly virulent community-acquired MRSA (USA300) strain was shown to be more sensitive to killing by neutrophils by both intracellular and extracellular mechanisms, as well as decreased survival in macrophages [21]. This MRSA *nos* mutant was also less virulent in a murine subcutaneous model of infection, displaying decreased abscess sizes and decreased recovered CFUs compared to the wild-type strain [21]. The UAMS-1 *nos*::*erm* mutant strain used in our current study was also less virulent than its wild-type counterpart in a murine sepsis model of infection (Fig. 8). A partial polar effect on *pdt* expression was observed in this mutant when grown in low-oxygen TSB cultures (Fig. 3B), therefore it is possible that the *in vivo* effects observed in the sepsis model were due, in part, to partial loss of *pdt* expression. Given the interest in both NOS and PDT as potential drug development targets [22], [23], our future studies will focus on assessing the individual contributions of *nos* and *pdt* to *S. aureus* virulence *in vivo* using non-polar deletion mutants.

As discussed above, *S. aureus* NOS clearly contributes to the oxidative stress response and virulence of this deadly pathogen. Considering the interest in this enzyme as a potential drug target, studies aimed at understanding the upstream regulators of both *nos* gene expression and saNOS enzyme activity are surprisingly lacking [22]. In the study presented here, we have confirmed that *S. aureus nos* is co-transcribed with the downstream *pdt* gene, encoding a prephenate dehydratase which catalyzes the formation of phenylpyruvate from prephenate in the phenylalanine biosynthesis pathway. Although saNOS contributes to oxidative stress resistance in *S. aureus*, we have demonstrated that expression of *nos-pdt* is upregulated in response to low-oxygen growth and is maximally expressed at late-exponential growth phase. These observations suggest that one or both of these enzymes are required for an as-yet unknown function in the normal physiology of this organism during low-oxygen growth. Although the genetic regulators of *nos-pdt* expression have not yet been identified, a search of the *S. aureus* microarray meta-database (SAMMD) [60] has revealed that *nos* expression was upregulated in *purH* [46], *lytS* [61], and *clpP* [62] mutants, as well as by challenge with H_2_O_2_ [40], and downregulated when challenged with chlorination [63], mupirocin [64], and various antimicrobial peptides [65].

The *S. aureus* PDT enzyme has also been postulated to be a potential drug target since it is involved in phenylalanine synthesis and is highly conserved among the staphylococci [23]. As such, the enzymatic activity of purified recombinant *S. aureus* PDT has been demonstrated and the crystal structure of this protein has been solved [23]. Although study of this gene in *S. aureus* has not been previously described, the data presented in this manuscript begin to fulfill this knowledge gap by confirming a role for saPDT as being required for growth in the absence of phenylalanine, as well as identifying low-oxygen growth as a signal that stimulates expression of *pdt in vitro*. RNA microarray analysis of *S. aureus* cultures previously revealed that *nos* and *pdt* were upregulated 2.5- and 2.2-fold, respectively, in H_2_O_2_-treated cultures [40]. Given these collective results, we hypothesized that saPDT, like saNOS, may also contribute to oxidative stress resistance, possibly by generating excess phenylpyruvate under these conditions. The α-keto acid anions, such as β-hydroxypyruvate and β-phenylpyruvate, can chemically react with H_2_O_2_, and have been proposed to act as antioxidants by nucleophilic attack of the mono-deprotonated peroxide species at the C-2 carbonyl group carbon center [42]. Furthermore, pyruvate, another α-keto acid, is commonly added as an H_2_0_2_-scavenger in certain cell culture media [66], and exogenous pyruvate is able to protect these cultured cells from H_2_O_2_-induced lysis [67]. However, our data suggests that saNOS is not required for activity of saPDT when participating in phenylalanine biosynthesis, and saPDT itself does not appear to contribute to hydrogen peroxide resistance under the *in vitro* conditions tested in this study (Figs. 4 and 5). Therefore the potential functional and/or biological relationship between these two enzymes remains a mystery and subject of our on-going investigations.

## Supporting Information

File S1 Contains the following files: **Figure S1.** Expression of the *nos-pdt* operon in *S. aureus* USA300 HOU as assessed by RNA-seq analysis. Data depicted in this figure was generated as part of a previously-published RNA-seq study [39], whereby RNA from 3 hour TSB cultures of strain USA300 HOU was subjected to Ion Torrent sequencing and resulting reads were aligned to the USA300 FPR genome. Arrows represent potential internal promoters and/or RNA processing sites. **Figure S2.** Detection of intracellular NO/RNS in low-oxygen cultures. UAMS-1 (pMK4), KR1010 (*nos*::*erm* mutant; pMK4), and KR1010 (complement; pMKnos) were grown as low-oxygen TSB cultures for 6.25 hours, followed by harvesting and resuspension in HBSS +5 µM DAF-FM dicaetate as described in Materials and Methods. Fluorescence (RFU) and OD_600_ readings were recorded after 90 minutes incubation in a microplate reader. Data represents the average of n = 3 independent experiments, error bars  =  SEM.
(PDF)
