# Supplementary material for: Contribution of the nos-pdt Operon to Virulence Phenotypes in Methicillin-Sensitive Staphylococcus aureus
Source: PLoS One. 2014 Oct 2;9(10):e108868. doi: 10.1371/journal.pone.0108868 (PMC4183505; doi:10.1371/journal.pone.0108868)
Supplement: File S1 — Contains the following files: Figure S1. Expression of the nos-pdt operon in S. aureus USA300 HOU as assessed by RNA-seq analysis. Data depicted in this figure was generated as part of a previously-published RNA-seq study [39], whereby RNA from 3 hour TSB cultures of strain USA300 HOU was subjected to Ion Torrent sequencing and resulting reads were aligned to the USA300 FPR genome. Arrows represent potential internal promoters and/or RNA processing sites. Figure S2. Detection of intracellular NO/RNS in low-oxygen cultures. UAMS-1 (pMK4), KR1010 (nos::erm mutant; pMK4), and KR1010 (complement; pMKnos) were grown as low-oxygen TSB cultures for 6.25 hours, followed by harvesting and resuspension in HBSS +5 µM DAF-FM dicaetate as described in Materials and Methods. Fluorescence (RFU) and OD600 readings were recorded after 90 minutes incubation in a microplate reader. Data represents the average of n = 3 independent experiments, error bars = SEM. (PDF) [file pone.0108868.s001.pdf]

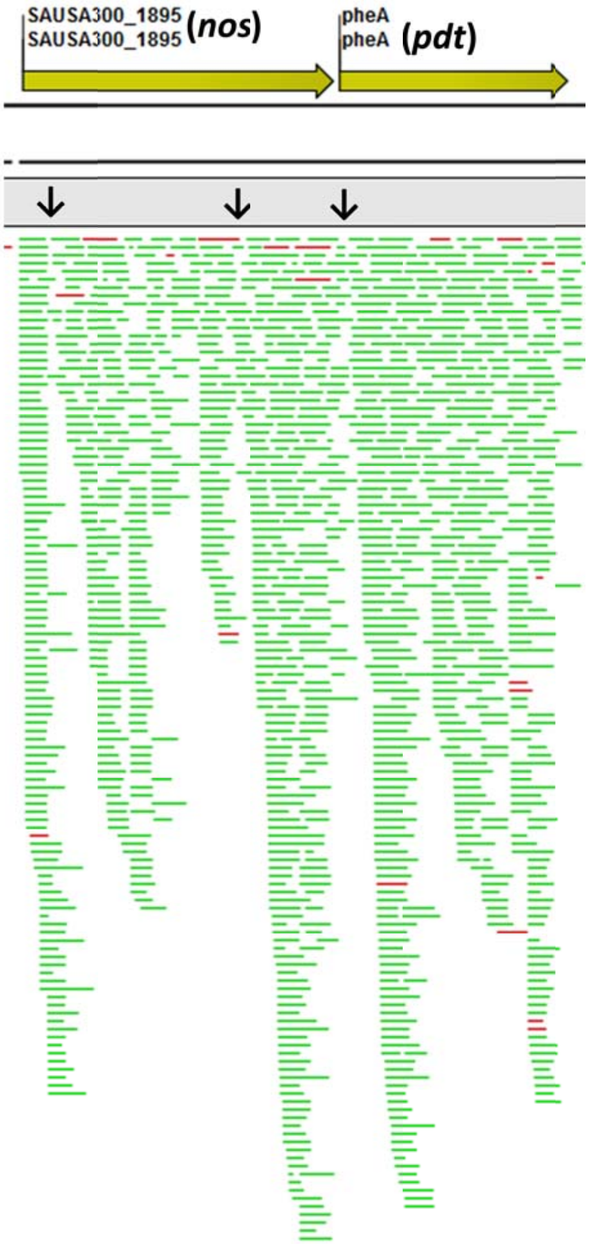

Figure S1. Expression of the *nos-pdt* operon in *S. aureus* USA300 HOU as assessed by RNA-seq analysis. Data depicted in this figure was generated as part of a previously-published RNA-seq study [39], whereby RNA from 3 hour TSB cultures of strain USA300 HOU was subjected to Ion Torrent sequencing and resulting reads were aligned to the USA300 FPR genome. Arrows represent potential internal promoters and/or RNA processing sites.

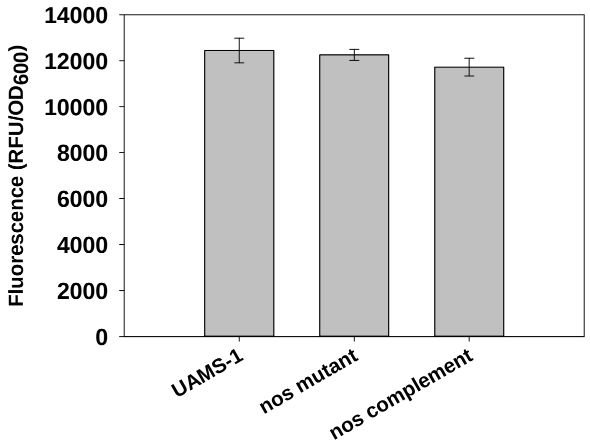

Figure S2. Detection of intracellular NO/RNS in low-oxygen cultures. UAMS-1 (pMK4), KR1010 (*nos*::*erm* mutant; pMK4), and KR1010 (complement; pMKnos) were grown as low-oxygen TSB cultures for 6.25 hours, followed by harvesting and resuspension in HBSS + 5 µM DAF-FM dicaetate as described in Materials and Methods. Fluorescence (RFU) and OD_{600} readings were recorded after 90 minutes incubation in a microplate reader. Data represents the average of n=3 independent experiments, error bars = SEM.
